# Left Ventricular Speckle Tracking-Derived Cardiac Strain and Cardiac Twist Mechanics in Athletes: A Systematic Review and Meta-Analysis of Controlled Studies

**DOI:** 10.1007/s40279-016-0644-4

**Published:** 2016-11-26

**Authors:** Alexander Beaumont, Fergal Grace, Joanna Richards, John Hough, David Oxborough, Nicholas Sculthorpe

**Affiliations:** 1 Institute of Clinical Exercise Physiology and Health Science, School of Science and Sport, University of the West of Scotland, Glasgow, UK; 2 Human Movement and Sports Science Group, Faculty of Health, Federation University Australia, Ballarat, VIC Australia; 3 Department of Sport Science and Physical Activity, Institute of Sport and Physical Activity Research, University of Bedfordshire, Bedford, UK; 4 Research Institute of Sport and Exercise Science, Liverpool John Moores University, Liverpool, UK

## Abstract

**Background:**

The athlete’s heart is associated with physiological remodeling as a consequence of repetitive cardiac loading. The effect of exercise training on left ventricular (LV) cardiac strain and twist mechanics are equivocal, and no meta-analysis has been conducted to date.

**Objective:**

The objective of this systematic review and meta-analysis was to review the literature pertaining to the effect of different forms of athletic training on cardiac strain and twist mechanics and determine the influence of traditional and contemporary sporting classifications on cardiac strain and twist mechanics.

**Methods:**

We searched PubMed/MEDLINE, Web of Science, and ScienceDirect for controlled studies of aged-matched male participants aged 18–45 years that used two-dimensional (2D) speckle tracking with a defined athlete sporting discipline and a control group not engaged in training programs. Data were extracted independently by two reviewers. Random-effects meta-analyses, subgroup analyses, and meta-regressions were conducted.

**Results:**

Our review included 13 studies with 945 participants (controls *n* = 355; athletes *n* = 590). Meta-analyses showed no athlete–control differences in LV strain or twist mechanics. However, moderator analyses showed greater LV twist in high-static low-dynamic athletes (*d* = –0.76, 95% confidence interval [CI] –1.32 to –0.20; *p* < 0.01) than in controls. Peak untwisting velocity (PUV) was greater in high-static low-dynamic athletes (*d* = –0.43, 95% CI –0.84 to –0.03; *p* < 0.05) but less than controls in high-static high-dynamic athletes (*d* = 0.79, 95% CI 0.002–1.58; *p* = 0.05). Elite endurance athletes had significantly less twist and apical rotation than controls (*d* = 0.68, 95% CI 0.19–1.16, *p* < 0.01; *d* = 0.64, 95% CI 0.27–1.00, *p* = 0.001, respectively) but no differences in basal rotation. Meta-regressions showed LV mass index was positively associated with global longitudinal (*b* = 0.01, 95% CI 0.002–0.02; *p* < 0.05), whereas systolic blood pressure was negatively associated with PUV (*b* = –0.06, 95% CI –0.13 to –0.001; *p* = 0.05).

**Conclusion:**

Echocardiographic 2D speckle tracking can identify subtle physiological differences in adaptations to cardiac strain and twist mechanics between athletes and healthy controls. Differences in speckle tracking echocardiography-derived parameters can be identified using suitable sporting categorizations.

## Key Points

**Table:** 

When athletes are not sufficiently categorized, exercise training appears to have little effect on cardiac strain and twist mechanics, but traditional and contemporary methods of sporting categorization can identify subtle differences in twist mechanics between athletes and controls.
Elite-level endurance athletes demonstrated reduced left ventricular twist and apical rotation, whereas competitive resistance athletes showed greater left ventricular twist and peak untwisting velocity than controls. Athletes also show greater untwisting rates than controls.
The lack of effect of exercise training on global longitudinal strain may suggest this parameter has potential for distinguishing pathological from physiological remodeling in athletes.

## Introduction

The concept that the hearts of athletes differ from those of non-athletes has aroused medical and public interest for more than a century [1]. In 1899, Henschen [2] used chest percussion to provide the first description of enlarged hearts in elite cross-country skiers. Progressive technological developments have furthered our understanding of how the heart undergoes morphological changes as a consequence of disease (pathological) or exercise training (physiological), with the latter becoming more widely known as ‘athlete’s heart’. Unlike the pathological processes that occur with heart disease, the athlete’s heart is an adaptive remodeling of cardiac tissue to accommodate the increased physiological demands of repetitive overload induced by exercise training [3, 4].

The first M-mode echocardiograms were performed by Edler and Hertz in 1953 [5, 6]. Since then, rapid technological advances have established two-dimensional (2D) echocardiography as a standard medical technique [5], identified left ventricular (LV) hypertrophy in athletes [7, 8], and allowed for comprehensive quantitative assessments of cardiac structure and function [9]. 2D speckle tracking echocardiography (STE) is a newer technology that facilitates the measurement of cardiac deformation by tracking acoustic speckle markers frame by frame within the ultrasound image [10, 11]. Although it was initially developed as an expansion of tissue Doppler imaging, it has the advantage of being relatively angle independent and enabling the assessment of movement within any direction of the imaging plane [12, 13]. STE enables assessment of the left ventricle as it undergoes a multi-planar process of deformation throughout the cardiac cycle [13] across three planes of motion: longitudinal, radial, and circumferential [14]. Furthermore, ‘twist mechanics’ can be determined. This concerns the cardiac twisting and untwisting that is mechanistically underpinned by the myocardial architecture and fiber arrangements and occurs, respectively, during systole and diastole [13]. Clockwise rotation at the base, and counter-clockwise rotation at the apex, of the myocardium constitute net LV twist; the directions are reversed upon diastole to produce untwisting, with the myocardium returning to its original shape and resting position [15].

Remodeling of cardiac tissue is considered to differ depending on the characteristic demands of a given sport, and has traditionally been studied between disciplines at polar ends of a scale, i.e., endurance versus resistance. Predominantly dynamic (endurance) sports such as distance running, Nordic skiing, and cycling require rapid and voluminous blood supply to working muscles. This is achieved via increased cardiac preload, which is typically considered to lead to eccentric ventricular hypertrophy, including chamber dilatation [16] and proportional increases in wall thickness [17]. Predominantly high-static (resistance) sports such as weightlifting, martial arts, and field throwing events induce elevations in intravascular pressure, which enhance afterload; adaptation is suggested to cause increased wall thickness in the absence of chamber dilatation, known as ‘concentric hypertrophy’ [18, 19]. However, some controversy exists concerning concentric morphology in resistance-trained athletes [20, 21].

Nevertheless, cardiac adaptations are relative to the degree of volume and pressure challenges induced by individual sports. Therefore, there is likely to be some overlap in the adaptations seen between individual sporting disciplines that have similar static and dynamic components. Accordingly, cardiac adaptations should be considered a relative concept [19, 22]. More recently, the traditional dichotomous classification of exercise has received criticism for its oversimplification [23]. Mitchell et al. [24] outlined a contemporary sporting categorization comprising a nine-box grid system that divides sports according to the dynamic (percentage maximum oxygen consumption) and static (percentage maximum voluntary contraction) components required and provides a more comprehensive division of sports. Detailed separation of athletes into their respective sporting groups may somewhat ameliorate the variability seen using the traditional classification to identify sport-specific cardiac adaptations.

In addition to the possibility of exercise-specific alterations in cardiac morphology, athletes in different sporting disciplines may also present alterations in systolic and diastolic function, including cardiac strain and twist mechanics. Numerous cross-sectional investigations have attempted to establish the deformation profiles of athletes compared with controls; however, evidence has been conflicting and no overall consensus regarding exercise effects has been found [10, 23, 25–38]. These problems are not resolved when comparing functional adaptations using the traditional dichotomous classification of endurance and resistance athletes versus controls [23, 32, 35, 37]. One study broadly utilized the contemporary framework by subdividing Olympic athletes into four groups according to their predominant training characteristics (skill, power, mixed discipline, endurance) [26]. Despite this, each group still included sports with an assortment of static and dynamic components, which resulted in heterogeneous samples and does not truly represent the ‘four corners’ of Mitchell’s classification. Consequently, use of a comprehensive classification system when studying LV strain and twist mechanics is still limited. Further to athlete type, training level may provide some explanation for the variations observed in athlete deformation profiles, particularly as differing structural and functional adaptations between elite and sub-elite athletes have been demonstrated [3]. However, any dose–response relationship between exercise training and STE-derived parameters is currently unknown. A recent review presented conflicting athlete–control differences, particularly in LV twist, and emphasized the need for additional data [39]. Clearly, more data are needed to enable exploration of alterations in athletes’ hearts due to chronic training. Categorizing sports into disciplines may aid in establishing potential modifications and exposing patterns in cardiac strain and twist mechanics.

To date, no meta-analysis has examined whether athlete–control differences occur in LV strain and twist mechanics. In light of this, we conducted a systematic review and meta-analysis to investigate potential sport-specific dependency using both traditional (endurance vs. resistance) and contemporary (Mitchell’s) classification systems and review how deformation responses in trained athletes differ from those in matched controls.

## Methods

The searching processes, study selection, data collection, analysis, and reporting of this systematic review and meta-analysis were conducted in accordance with PRISMA (Preferred Reporting Items for Systematic review and Meta-Analyses) guidelines [40]. The primary research question for this analysis was “Are there differences in STE characteristics of athletes when grouped using Michell’s nine-group model or when using a traditional endurance/resistance exercise model?”

A further research question concerned assessing the degree to which training status (elite vs. competitive) influenced the deformation characteristics of athletes.

### Information Sources and Search strategy

**Fig. 1:**
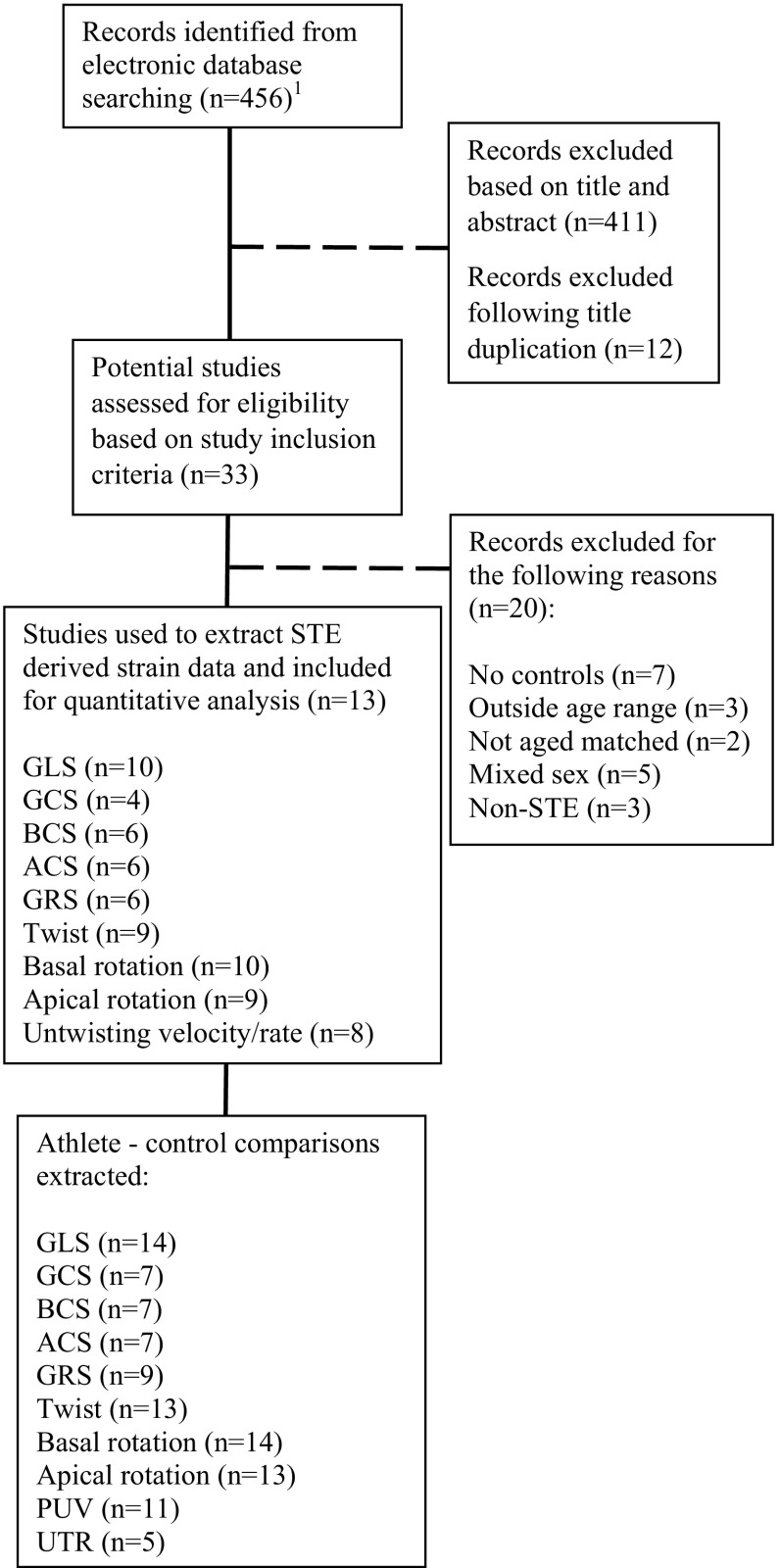
Schematic of literature searching and filtration process used for identification of eligible studies. *ACS* apical circumferential strain, *BCS* basal circumferential strain, *GCS* global circumferential strain, *GLS* global longitudinal strain, *GRS* global radial strain, *n* number of studies, *PUV* peak untwisting velocity, *STE* speckle tracking echocardiography, *UTR* untwisting rate. ^1^The electronic search was conducted as follows: echocardiography[Title/Abstract] OR ultrasound[Title/Abstract] OR left ventricular[Title/Abstract] OR two dimensional[Title/Abstract] NOT right ventricular[Title/Abstract] AND strain[Title/Abstract] OR speckle tracking[Title/Abstract] OR deformation[Title/Abstract] OR mechanics[Title/Abstract] AND athletes[Title/Abstract] OR exercise[Title/Abstract] OR trained[Title/Abstract] AND Journal Article[pytp] AND “2005/01/01”[PDAT]: “2016/01/01[PDAT] AND “humans”[MeSH Terms] AND English[lang]

We conducted an electronic database search to identify 2D STE studies investigating LV strain in athletic men. We searched PubMed/MEDLINE (abstract/title), Web of Science (title only), and ScienceDirect (abstract/title/keywords) until January 2016 to identify studies published from the earliest possible date to 1 January 2016. Further filters were applied so only English language journal articles concerning human subjects were retrieved. Review articles, meta-analyses, and longitudinal studies were excluded. Search terms associated with the athlete’s heart were used in conjunction with Boolean operators (Fig. 1). We extended the initial search via cross-referencing, and added articles we knew of that were not initially found during the systematic search.

### Inclusion Criteria

To ensure we could carry out quantitative analysis, inclusion criteria were as follows: (1) participants were male; (2) subjects were aged 18–45 years; (3) the study included an age-matched cohort; (4) subjects were athletes from a stated sporting discipline; (5) the study used an observational design; (6) the study used 2D STE; (7) the study included a control group not engaged in training programs; and (8) the study investigated at least one or more LV strain parameter. Only males were included because current knowledge indicates that cardiac strain may be sex dependent [26, 41, 42]. Likewise, twist mechanics are known to be affected by age [43–46]. Therefore, we opted to employ a broad age range to maximize article inclusion while attempting to limit potential confounding factors.

### Study Selection and Data Extraction

Literature searching and study selections were performed independently by the authors AB and NS. AB extracted all associated data from each investigation and entered them into a spreadsheet (Microsoft^®^ Excel 2016, Microsoft Corporation, Redmond, WA, USA). Nine measures were obtained in total, including 5 strain measures, as follows: (1) global longitudinal strain (GLS); (2) basal circumferential strain (BCS); (3) apical circumferential strain (ACS); (4) global circumferential strain (GCS); (5) global radial strain (GRS); and 4 measures of LV twist mechanics (6) basal rotation; (7) apical rotation; (8) twist; and (9) untwisting rate/velocity. GLS was determined as the average segmental strain from the apical four-chamber view, a combination of apical four- and two-chamber views, or apical four-, two-, and three-chamber views. We used basal and medium apical segmental longitudinal strain data to determine GLS when available. When specified, GCS was the segmental average strain obtained from the short-axis mid-level or the combination of apical, mid, and basal levels. Unless an article stated otherwise, we assumed the ACS and BCS were the average of the automatically generated six segments. GRS was considered the segmental average strain of the mid-level short-axis view or a combination of the apical, mid, and basal levels. Since we did not use apical and basal radial strain as independent parameters within this meta-analysis, we used them to determine GRS. Data were extracted for twist from studies that reported a single time point at peak or end systole (aortic valve closure). Studies often used untwisting rate (UTR) to refer to peak untwisting velocity (PUV) [32, 34, 47], with peak UTR defined as the PUV occurring during early diastole [30, 48]. UTR has also been used to describe the rate of untwisting occurring during the earliest phases of diastole at timing events prior to mitral valve opening (MVO) [10, 37]. As terms are often used interchangeably, for the purposes of this meta-analysis, we separated the untwisting indices: peak untwisting markers were categorised as ‘PUV’—the largest negative deflection following peak twist velocity [49], whereas untwist (°/sec) determined at or prior to MVO were categorized as ‘UTR’ when clearly detailed. Data were extrapolated from text, tables, and figures. When torsion/time graphs were presented, peak measures during systole (0–100% systole) were obtained.

Study means ± standard deviation (SD) were recorded for all variables; however, where studies reported the standard error of the mean (SEM), we applied a manual conversion using the formula SD = SEM *√*N*, where *N* is the number of participants. Age and cardiac morphology were recorded along with covariates associated with the hemodynamic loading exerted upon the myocardium: heart rate (HR), systolic blood pressure (SBP), diastolic blood pressure (DBP), and left ventricular mass index (LVMi).

### Data Grouping

All athlete grouping was conducted by one author (AB) then verified by a second author (NS). When a single sporting discipline was reported, each athlete sample was allocated an assigned group based on Mitchell’s classification [24]:

1. A1 (low dynamic, low static);
2. A2 (low dynamic, moderate static);
3. A3 (low dynamic, high static);
4. B1 (moderate dynamic, low static);
5. B2 (moderate dynamic, moderate static);
6. B3 (moderate dynamic, high static);
7. C1 (high dynamic, low static);
8. C2 (high dynamic, moderate static);
9. C3 (high dynamic, high static).

We used an additional separate categorization using a traditional method to divide sports that were either predominantly endurance based or resistance based. We applied further subdivisions on the basis of athlete training level: either ‘elite’ or ‘competitive’ performers. Elite athletes included those who were described as elite or who participated in professional competitions or at a national/international level. Competitive athletes were ‘amateur’, ‘competitive’, or ‘highly trained’ subjects.

**Fig. 2:**
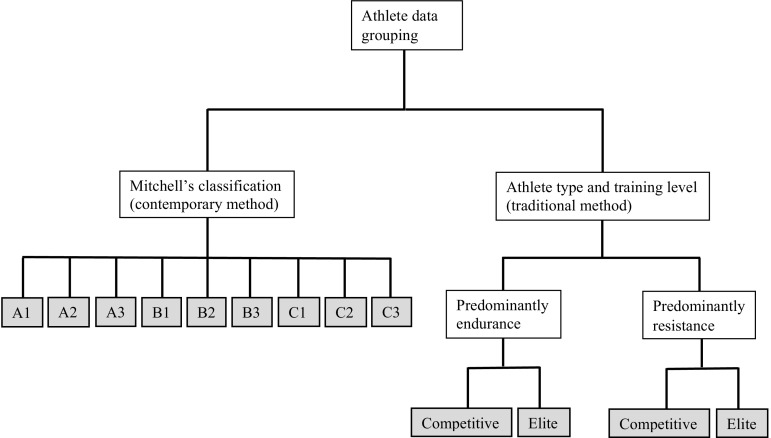
Model of athlete grouping using the contemporary Mitchell’s classification and a traditional dichotomous classification with additional grouping based on athlete training level. *Filled boxes* indicate endpoints of the classifications; athletes were allocated into one group for each method. *A1* low dynamic, low static, *A2* low dynamic, moderate static, *A3* low dynamic, high static, *B1* moderate dynamic, low static, *B2* moderate dynamic, moderate static, *B3* moderate dynamic, high static, *C1* high dynamic, low static, *C2* high dynamic, moderate static, *C3* high dynamic, high static

Therefore, athletes were allocated into one of four potential groups (elite endurance, competitive endurance, elite resistance, competitive resistance). Figure 2 illustrates the model used in this meta-analysis for the athlete data grouping according to Mitchell’s classification (contemporary) and the traditional dichotomous model.

### Statistical Analyses

All data analyses were performed using Comprehensive Meta-Analysis (Biostat: V 2.2.064, Englewood, NJ, USA). Pooled data were used to complete the meta-analysis using a random-effects model to investigate athlete–control differences. Standardized difference in means (Cohen’s d)/effect sizes were calculated for each individual study, and a summary with overall effect size recorded for each group of studies. Effect sizes in a positive direction indicated greater LV mechanics in controls, whereas a negative direction identified greater mechanics in athletes. Moderator analyses were performed by dividing studies using categorical moderator variables (Mitchell’s classification and traditional categorization with training level) as separate analyses. Using continuous moderator variables (age, HR, SBP, DBP, LVMi), we conducted multiple meta-regressions using methods of moments to establish relationships with LV mechanics. Heterogeneity was reported using Cochran’s *Q* and *I* ^2^ statistic (the percentage of total variation between studies due to heterogeneity rather than chance) and classed as low, moderate, and high at 25, 50, and 75%, respectively [50]. Publication bias was addressed using funnel plots, followed by Egger’s regression intercept [51] to test for asymmetry; however, caution in intepreting the results is recommended as there were fewer than 10 studies in the meta-analysis [52]. Statistical significance was granted at *p* ≤ 0.05.

## Results

### Search Outcome

The literature search resulted in 456 records; 411 of these were excluded after title and abstract screening, mainly because they lacked an athletic focus. The remaining articles were exported and 12 duplicates were removed. The full texts of potential articles were examined for eligibility, and 20 investigations were removed because they did not include a control group (*n* = 7); group means were outside the age range (*n* = 3); athlete and control groups were not aged matched (*n* = 2); samples included both sexes (*n* = 5), or deformation was not measured with STE (*n* = 3). Subsequently, 13 studies including 945 participants (590 athletes and 355 controls) met the inclusion criteria and were used for statistical analyses [10, 23, 28, 30–34, 36–38, 53, 54].

Strain variables were identified from the 13 studies used for analysis: GLS (*n* = 10) [23, 28, 31–34, 36, 37, 53, 54], BCS (*n* = 6) [28, 31–34, 53], ACS (*n* = 6) [28, 31–34, 53], GCS (*n* = 4) [23, 28, 37, 54], GRS (*n* = 6) [23, 28, 31, 37, 53, 54], twist (*n* = 9) [10, 28, 30–34, 37, 53], basal rotation (*n* = 10) [10, 28, 30–34, 37, 38, 53], apical rotation (*n* = 9) [28, 30–34, 37, 38, 53], and untwisting velocity/rate (*n* = 8) [10, 30–34, 37, 53]. Where more than one athlete–control comparison was reported, this was documented as a separate comparison whereby the control *n* was divided by the number of comparisons available, leading to GLS (*n* = 14), BCS (*n* = 7), ACS (*n* = 7), GCS (*n* = 7), GRS (*n* = 9), twist (*n* = 13), basal rotation (*n* = 14), apical rotation (*n* = 13), PUV (*n* = 11), and UTR (*n* = 5) (Fig. 1). Tables 1 and 2, respectively, summarize LV strain and twist mechanics data for control and athlete groups and Table 3 presents all athlete–control comparisons and heterogeneity for the strain measures GLS, GCS, ACS, BCS, and GRS and basal and apical rotations.

**Table 1:** Summary of studies assessing left ventricular strain

Study (year of publication)	Study group (Mitchell classification, training level)	*n*	Age (years)	GLS (%)	GCS (%)	BCS (%)	ACS (%)	GRS (%)	Overall findings				
									GLS	GCS	BCS	ACS	GRS
Santoro et al. (2014) [32]	Control	17	24.5 ± 3	–17.7 ± 2.8	–	–16.5 ± 3.4	–27.8 ± 5.6	–					
	Cyclists (C3, E)	33	24.0 ± 3	–16.5 ± 1.7	–	–14.6 ± 3.0	–21.6 ± 4.1	–	↔	–	↔	↓	–
	Weightlifters (A3, C)	36	24.6 ± 5	–16.6 ± 2.1	–	–16.7 ± 2.4	–26.8 ± 7.7	–	↔	–	↔	↔	–
Santoro et al. (2014) [34]	Control	17	40.4 ± 9.2	–20.1 ± 2.3	–	–17.1 ± 3.5	–27.8 ± 1.5	–					
	Water polo players (C2, E)	45	39.2 ± 6.8	–19.2 ± 5.0	–	–16.4 ± 3.2	–25.9 ± 4.3	–	↔	–	↔	↔	–
Santoro et al. (2015) [33]	Control	95	32.0 ± 6.0	–19.3 ± 2.8	–	–16.1 ± 4.2	–23.4 ± 6.4	–					
	Swimmers (C2, C)	125	30.0 ± 9.0	–20.4 ± 2.5	–	–17.6 ± 5.8	–22.7 ± 7.2	–	↑	–	↑	↔	–
Nottin et al. (2008) [31]	Control	23	24.6 ± 4.6	–19.5 ± 2.2	–	–16.2 ± 3.4	–18.6 ± 4.1	46.9 ± 14.4					
	Cyclists (C3, E)	16	22.6 ± 5.4	–19.2 ± 1.9	–	–16.0 ± 3.5	–18.1 ± 2.5	42.2 ± 11.2	↔	–	↔	↔	↔
Szauder et al. (2015) [23]	Control	15	27.0 ± 3.0	–24.1 ± 3.0	–26.4 ± 2.7	–	–	44.1 ± 4.5					
	(Ultra)marathoners (C1, E)	24	27.0 ± 3.0	–19.4 ± 3.4	–26.6 ± 3.8	–	–	42.5 ± 5.5	↓	↔	–	–	↔
	Body builders (B3, C)	14	27.0 ± 3.0	–23.3 ± 2.1	–22.4 ± 4.3	–	–	44.2 ± 8.2	↔	↓	–	–	↔
Vitarelli et al. (2013) [37]	Control	35	28.3 ± 11.4	–20.3 ± 2.6	–24.7 ± 3.4	–	–	48.9 ± 9.7					
	Marathoners (C1, C)	35	28.7 ± 10.7	–21.7 ± 2.6	–22.9 ± 3.3	–	–	46.9 ± 9.4	↔	↔	–	–	↔
	Power lifters (A3, C)	35	30.3 ± 9.4	–22.5 ± 2.4	–24.1 ± 2.7	–	–	49.6 ± 8.5	↑	↔	–	–	↔
	Martial artists (A3, C)	35	29.4 ± 9.8	–21.6 ± 2.2	–22.6 ± 3.6	–	–	47.5 ± 8.7	↔	↔	–	–	↔
Stefani et al. (2008) [36]	Control	25	25.0 ± 2.6	–19.4 ± 5.2	–	–	–	–					
	Soccer players (C1, E)	25	26.0 ± 3.5	–18.6 ± 3.3	–	–	–	–	↔	–	–	–	–
Galderisi et al. (2010) [28]	Control	19	28.5 ± 6.6	–21.1 ± 2.0	–17.6 ± 2.9	–16.7 ± 2.7	–17.8 ± 2.9	46.4 ± 15.8					
	Rowers (C3, E)	22	27.7 ± 8.4	–22.2 ± 2.7	–17.7 ± 2.5	–16.8 ± 2.4	–17.8 ± 2.6	47.6 ± 19.1	↔	↔	↔	↔	↔
Cote et al. (2013) [53]	Control	10	28.5 ± 5.9	–19.0 ± 2.9	–	–16.3 ± 5.3	–26.4 ± 10.2	25.7 ± 9.6					
	Cyclists (C3, C)	11	33.0 ± 5.6	–18.5 ± 2.1	–	–16.6 ± 4.3	–25.9 ± 10.7	33.9 ± 12.8	↔	–	↔	↔	↔
Donal et al. (2011) [54]	Control	27	26.2 ± 3.1	–17.7 ± 1.6	–15.9 ± 8.5	–	–	44.1 ± 11.0					
	Cyclists (C3, C)	18	25.2 ± 5.0	–17.0 ± 1.3	–17.4 ± 3.3	–	–	38.7 ± 7.8	↔	↔	–	–	↔

Data are mean ± standard deviation unless otherwise indicated

*A3* low dynamic, high static, *ACS* apical circumferential strain, *B3* moderate dynamic, high static, *BCS* basal circumferential strain, *C* competitive athletes, *C1* high dynamic, low static, *C2* high dynamic, moderate static, *C3* high dynamic, high static, *E* elite athletes, *GCS* global circumferential strain, *GLS* global longitudinal strain, *GRS* global radial strain, *n* participant number, ↑ indicates significantly greater in athletes, ↓ indicates significantly less in athletes, ↔ indicates no athlete-control differences

**Table 2:** Summary of studies assessing left ventricular twist mechanics

Study (year of publication)	Study group (Mitchell classification, training level)	*n*	Age (years)	Systolic parameters			Diastolic parameters		Overall findings				
				Brot (°)	Arot (°)	Twist (°)	PUV (°/sec)	UTR (°/sec)					
									Brot	Arot	Twist	PUV	UTR
Santoro et al. (2014) [32]	Control	17	24.5 ± 3	–8.5 ± 7.4	6.3 ± 2.8	10.0 ± 3.1	–103.3 ± 29.3	–					
	Cyclists (C3, E)	33	24.0 ± 3	–6.4 ± 2.1	4.2 ± 1.9	6.2 ± 1.1	–67.3 ± 22.9	–	↔	↓	↓	↓	–
	Weightlifters (A3,C)	36	24.6 ± 5	–5.8 ± 2.3	7.6 ± 5.4	12.0 ± 2.1	–122.5 ± 52.8	–	↔	↔	↑	↔	–
Santoro et al. (2014) [34]	Control	17	40.4 ± 9.2	–4.3 ± 1.2	6.5 ± 1.1	10.3 ± 2.4	–108.4 ± 39.5	–					
	Water polo players (C2, E)	45	39.2 ± 6.8	–4.9 ± 1.6	6.1 ± 0.3	8.8 ± 3.6	–79.9 ± 35.9	–	↔	↓	↔	↓	–
Santoro et al. (2015) [33]	Control	95	32.0 ± 6.0	–6.7 ± 3.8	8.2 ± 4.0	12.2 ± 5	–96.2 ± 48.7	–					
	Swimmers (C2, C)	125	30.0 ± 9.0	–5.9 ± 3.4	6.1 ± 3.4	9.0 ± 3.8	–94.5 ± 40.3	–	↔	↓	↓	↔	–
Nottin et al. (2008) [31]	Control	23	24.6 ± 4.6	–4.8 ± 3.2	4.0 ± 2.9	9.2 ± 3.2	–72.9 ± 20.7	–					
	Cyclists (C3, E)	16	22.6 ± 5.4	–5.2 ± 2.4	1.7 ± 1.9	6.0 ± 1.8	–68.2 ± 33.5	–	↔	↓	↓	↔	–
Vitarelli et al. (2013) [37]	Control	35	28.3 ± 11.4	–6.7 ± 2.3	10.1 ± 3.6	14.6 ± 4.3	–78.9 ± 15.0	–61.7 ± 24.0					
	Marathoners (C1, C)	35	28.7 ± 10.7	–7.7 ± 2.2	14.2 ± 4.3	21.5 ± 5.2	–93.9 ± 21.0	–94.2 ± 29.0	↔	↑	↑	↑	↑
	Power lifters (A3,C)	35	30.3 ± 9.4	–6.8 ± 1.9	10.8 ± 3.7	15.8 ± 4.5	–83.1 ± 16.0	–64.2 ± 23.0	↔	↔	↔	↔	↔
	Martial artists (A3,C)	35	29.4 ± 9.8	–7.6 ± 2.4	13.8 ± 3.9	20.8 ± 5.4	–92.2 ± 22.0	–80.6 ± 31.0	↔	↑	↑	↔	↔
Cote et al. (2013) [53]	Control	10	28.5 ± 5.9	–11.7 ± 21.3	12.9 ± 5.2	17.8 ± 6.2	–139.9 ± 44.5	–					
	Cyclists (C3, C)	11	33.0 ± 5.6	–5.8 ± 1.7	12.2 ± 3.9	17.4 ± 4.9	–108.7 ± 33.0	–	↔	↔	↔	↔	–
Galderisi et al. (2010) [28]	Control	19	28.5 ± 6.6	–3.7 ± 0.5	6.2 ± 1.4	9.7 ± 1.8	–	–					
	Rowers (C3, E)	22	27.7 ± 8.4	–2.9 ± 1.5	6.1 ± 2.3	9.2 ± 2.0	–	–	↓	↔	↔	–	–
Kovacs et al. (2014) [10]	Control	13	30.0 ± 5.0	–2.1 ± 1.0	–	6.0 ± 2.2	–						
	Kayak, canoe, and rowers (C3, E)	28	26.0 ± 8.0	–2.7 ± 1.2	–	6.4 ± 2.1	–	AVC: –7.4 ± 9.3 –12.2 ± 8.8 MVO: –23.2 ± 8.2 –32.7 ± 12.7	↔	–	↔	–	AVC: ↔ MVO: ↑
Maufrais et al. (2014) [30]	Control	30	21.3 ± 3.0	–5.6 ± 2.5	5.6 ± 2.9	8.9 ± 3.3	–66.7 ± 27.5	–					
	Marathoners, triathletes, and cyclists (ND, E)	25	23.0 ± 2.0	–4.9 ± 2.2	4.9 ± 2.0	8.0 ± 3.0	–76.7 ± 34.0	–	↔	↔	↔	↔	–
Maufrais et al. (2014) [30]	Control	19	38.0 ± 5.0	–5.9 ± 3.5	7.2 ± 3.4	11.5 ± 4.5	–69.5 ± 29.0	–					
	Marathoners, triathletes and cyclists (ND, E)	46	38.0 ± 5.0	–4.2 ± 1.5	6.2 ± 2.6	8.5 ± 3.0	–74.9 ± 24.1	–	↓	↔	↓	↔	–
Zocalo et al. (2008) [38]	Control	10	27.0 ± 6.0	–7.4 ± 0.9	6.9 ± 2.5	–	–	–					
	Soccer players (C1, E)	17	25.0 ± 5.0	–2.7 ± 2.8	3.1 ± 1.8	–	–	–	↓	↓	–	–	–

Data are mean ± standard deviation unless otherwise indicated

*A3* low dynamic, high static, *Arot* apical rotation, *AVC* aortic valve closure, *Brot* basal rotation, *C1* high dynamic, low static, *C2* high dynamic, moderate static, *C3* high dynamic, high static, *E* elite athletes, *C* competitive athletes, *MVO* mitral valve opening, *n* participant number, *ND* not differentiated, *PUV* peak untwisting velocity, *UTR* untwisting rate, ↑ indicates significantly greater in athletes, ↓ indicates significantly less in athletes, ↔ indicates no athlete–control differences

**Table 3:** Meta-analyses of athlete-control comparisons for left ventricular strain and twist mechanics

Parameter	Number of studies	*d*	95% CI	*p* value	Heterogeneity		*p* value
					Cochran’s *Q*	*I* ^2^ statistic (%)	
Global longitudinal strain							
Overall	14	0.04	–0.25 to 0.33	0.80	39.75	67.30	**<0.001**
Mitchell classification							
A3	3	–0.34	–1.13 to 0.45	0.40	7.29	72.58	**0.03**
B3	1	0.33	–0.58 to 1.24	0.48	–	–	–
C1	3	0.32	–0.67 to 1.30	0.53	12.24	83.66	**0.002**
C2	2	–0.16	–0.76 to 0.44	0.61	3.84	73.93	**0.05**
C3	5	0.17	–0.21 to 0.55	0.38	6.16	35.03	0.19
Between	–	–	–	–	10.23	–	**0.04**
Athlete and trainin g level							
Endurance_competitive_	4	–0.13	–0.60 to 0.35	0.61	8.58	65.05	**0.04**
Endurance_elite_	6	0.29	–0.14 to 0.72	0.18	12.91	61.26	**0.02**
Resistance_competitive_	4	–0.20	–0.86 to 0.47	0.56	9.24	67.53	**0.03**
Between	–	–	–	–	9.03	–	**0.01**
Global circumferential strain							
Overall	7	0.24	–0.07 to 0.54	0.12	7.96	24.60	0.24
Mitchell classification							
A3	2	0.39	–0.09 to 0.87	0.11	0.62	0.00	0.43
B3	1	1.03	0.08 to 1.99	**0.04**	–	–	–
C1	2	0.29	–0.29 to 0.87	0.33	1.27	21.03	0.26
C3	2	–0.13	–0.56 to 0.30	0.56	0.17	0.00	0.68
Between	–	–	–	–	5.90	–	0.12
Athlete and training level							
Endurance_competitive_	2	0.15	–0.59 to 0.89	0.67	2.76	63.77	0.10
Endurance_elite_	2	–0.04	–0.53 to 0.44	0.86	0.00	0.00	0.97
Resistance_competitive_	3	0.52	0.09 to 0.95	**0.02**	2.01	0.57	0.37
Between	–	–	–	–	3.19	–	0.20
Apical circumferential strain							
Overall	7	0.29	–0.02 to 0.59	0.06	10.89	44.91	0.09
Mitchell classification							
A3	1	0.14	–0.63 to 0.90	0.73	–	–	–
C2	2	0.22	–0.14 to 0.59	0.23	1.60	37.41	0.21
C3	4	0.37	–0.24 to 0.99	0.23	8.78	65.82	**0.03**
Between	–	–	–	–	0.52	–	0.77
Athlete and training level							
Endurance_competitive_	2	0.10	–0.16 to 0.35	0.46	0.02	0.00	0.90
Endurance_elite_	4	0.47	–0.07 to 1.01	0.09	8.41	64.31	**0.04**
Resistance_competitive_	1	0.14	–0.63 to 0.90	0.73	–	–	–
Between	–	–	–	–	2.47	–	0.29
Basal circumferential strain							
Overall	7	–0.05	–0.27 to 0.18	0.68	6.92	13.35	0.33
Mitchell classification							
A3	1	–0.08	–0.84 to 0.69	0.84	–	–	–
C2	2	–0.10	–0.58 to 0.38	0.68	2.53	60.47	0.11
C3	4	0.13	–0.22 to 0.48	0.47	2.14	0.00	0.54
Between	–	–	–	–	2.25	–	0.32
Athlete and training level							
Endurance_competitive_	2	–0.27	–0.53 to –0.01	**0.04**	0.26	0.00	0.61
Endurance_elite_	4	0.18	–0.14 to 0.50	0.26	1.95	0.00	0.58
Resistance_competitive_	1	–0.08	–0.84 to 0.69	0.84	–	–	**–**
Between	–	–	–	–	4.72	–	0.10
Global radial strain							
Overall	9	0.13	–0.11 to 0.36	0.29	6.97	0.00	0.54
Mitchell classification							
A3	2	0.04	–0.44 to 0.51	0.89	0.24	0.00	0.62
B3	1	–0.01	–0.92 to 0.89	0.98	–	–	–
C1	2	0.25	–0.26 to 0.76	0.34	0.03	0.00	0.86
C3	4	0.09	–0.40 to 0.58	0.71	6.24	51.91	0.10
Between	9	–	–	–	0.46	–	0.93
Athlete and training level							
Endurance_competitive_	3	0.08	–0.59 to 0.75	0.82	5.40	62.98	0.07
Endurance_elite_	3	0.17	–0.22 to 0.56	0.38	0.99	0.00	0.61
Resistance_competitive_	3	0.02	–0.39 to 0.44	0.91	0.25	0.00	0.88
Between	–	–	–	–	0.32	–	0.85
Basal rotation							
Overall	14	0.22	–0.06 to 0.51	0.13	40.17	67.63	**<0.001**
Mitchell classification							
A3	3	0.08	–0.54 to 0.69	0.81	4.55	56.04	0.10
C1	2	0.75	–1.65 to 3.15	0.54	17.32	94.23	**<0.001**
C2	2	–0.04	–0.64 to 0.57	0.91	3.84	73.95	**0.05**
C3	5	0.18	–0.29 to 0.65	0.45	8.99	55.53	0.06
Between	–	–	–	–	0.92	–	0.82
Athlete and training level							
Endurance_competitive_	3	0.08	–0.38 to 0.52	0.74	3.78	47.02	0.15
Endurance_elite_	8	0.36	–0.12 to 0.83	0.14	30.56	77.09	**<0.001**
Resistance_competitive_	3	0.08	–0.54 to 0.69	0.81	4.55	56.04	0.10
Between	–	–	–	–	1.28	–	0.53
Apical rotation							
Overall	13	0.25	–0.10 to 0.60	0.17	52.67	77.22	**<0.001**
Mitchell classification							
A3	3	–0.47	–0.96 to 0.02	0.06	2.88	30.62	0.24
C1	2	0.39	–2.35 to 3.13	0.78	22.90	95.63	**<0.001**
C2	2	0.59	0.34 to 0.83	**<0.001**	0.05	0.00	0.83
C3	4	0.52	0.03 to 1.02	**0.04**	5.64	46.84	0.13
Between	–	–	–	–	21.16	–	**<0.001**
Athlete and training level							
Endurance_competitive_	3	–0.06	–1.06 to 0.94	0.91	17.43	88.52	**<0.001**
Endurance_elite_	7	0.64	0.27 to 1.00	**0.001**	13.71	56.24	**0.03**
Resistance_competitive_	3	–0.47	–0.96 to 0.02	0.06	2.88	30.62	0.24
Between	–	–	–	**–**	18.65	–	**<0.001**

Bold values indicate statistical significance

*A3* high static, low dynamic, *B3* high static, moderate dynamic, *C1* high dynamic, low static, *C2* high dynamic, moderate static, *C3* high dynamic, high static, *CI* confidence interval

### Global Longitudinal Strain

GLS was analysed overall and in C3 (high dynamic, high static), B3 (moderate dynamic, high static), A3 (low dynamic, high static), C2 (high dynamic, moderate static), C1 (high dynamic, low static), elite endurance, competitive endurance, and competitive resistance athlete groups compared with controls. No athlete–control differences existed for GLS overall, following sporting categorization or training level. Overall, there was significant heterogeneity with moderate inconsistency. Mitchell’s sporting categorization showed heterogeneity was significant in A3, C1 and C2 groups, with inconsistency considered low in C3 and B3, moderate in A3 and C2, and high in C1. Significant heterogeneity was found between sporting groups. Traditional categorization showed heterogeneity was significant and inconsistency was moderate in all groups. Between-group heterogeneity statistically differed. The funnel plot revealed three studies that lay outside of the standard error (SE) funnel, suggesting asymmetry. However, Egger’s test did not significantly confirm this visualization of asymmetry; the intercept was 2.40 (95% confidence interval [CI] two-tailed, –0.14 to 4.94; two-tailed *p* = 0.06).

### Circumferential Strain

#### Global

GCS was analysed overall and in A3 (low dynamic, high static), C1 (high dynamic, low static), C3 (high dynamic, high static), B3 (moderate dynamic, high static), elite endurance, competitive endurance, and competitive resistance athlete groups compared with controls. Overall, no athlete–control differences existed for GCS. Between-study heterogeneity was non-significant and inconsistency was low. There were no differences between athletes and controls in the A3, C1, and C3 groups, whereas B3 athletes showed lower GCS than controls. All groups showed non-significant heterogeneity with low inconsistency. Non-significant heterogeneity was found between groups. Traditional categorization showed competitive resistance athletes had significantly less GCS than controls, whereas no differences were seen in either endurance group. Heterogeneity was non-significant in all groups, with low inconsistency in endurance elite and resistance competitive groups but moderate inconsistency in endurance competitive, with non-significance between groups. Visual inspection of the funnel plot showed no studies were outside of the funnel, which confirmed no asymmetry by Egger’s regression (intercept = 4.72; 95% CI two-tailed –2.05 to 11.49; two-tailed *p* = 0.13).

#### Basal

BCS was analysed overall and in A3 (low dynamic, high static), C3 (high dynamic, high static), C2 (high dynamic, moderate static), elite endurance, competitive endurance, and competitive resistance athlete groups compared with controls. No athlete–control differences were found for BCS overall or with Mitchell’s classification. Overall, between-study heterogeneity was non-significant and inconsistency was low. Between-study heterogeneity was non-significant within all groups, inconsistency was low in A3 and C3 but moderate in C2. Between-group heterogeneity was non-significant. Traditional categorization showed endurance competitive athletes had significantly greater BCS than controls; no differences were found in elite endurance or competitive resistance athletes. Study-to-study heterogeneity in all groups was non-significant with low inconsistency. There was no significant heterogeneity between groups. The funnel plot showed no studies were outside of the funnel; however, weighting was greater to the right side. Asymmetry was confirmed by Egger’s regression test (intercept = 1.79; 95% CI two-tailed –0.03 to 3.62; two-tailed *p* = 0.05).

#### Apical

ACS was analysed overall and in C3 (high dynamic, high static), A3 (low dynamic, high static), C2 (high dynamic, moderate static), elite endurance, competitive endurance, and competitive resistance athlete groups compared with controls. ACS did not significantly differ between athletes and controls overall, using Mitchell’s or traditional categorization or training level. Overall, study-to-study heterogeneity was non-significant with low inconsistency. Within-group heterogeneity was non-significant with low inconsistency in A3 and C2, but significant with moderate inconsistency in C3. Non-significant heterogeneity was found between groups. Heterogeneity within the endurance competitive group was non-significant with low inconsistency. In contrast, the endurance elite group showed significant heterogeneity accompanied by moderate inconsistency. In addition, no significant between-group heterogeneity was found. One study fell outside the funnel plot. In contrast, Egger’s regression suggested no asymmetry (intercept = 1.26; 95% CI two-tailed –1.79 to 4.31; two-tailed *p* = 0.34).

### Global Radial Strain

GRS was analysed overall and in A3 (low dynamic, high static), B3 (moderate dynamic, high static), C1 (high dynamic, low static), C3 (high dynamic, high static), elite endurance, competitive endurance, and competitive resistance athlete groups compared with controls. The overall athlete–control effect indicated no differences. Between-study inconsistency was considered low with non-significant heterogeneity. Similarly, with Mitchell’s classification, no sporting discipline group showed athlete–control differences. Within-group heterogeneity was non-significant in all cases, inconsistency was low in A3, B3, and C1 but moderate in C3. Between-group heterogeneity was non-significant. Traditional categorization with training level had no effect on the athlete–control differences, with non-significant heterogeneity in all groups with low inconsistency in elite endurance and competitive resistance groups but moderate in competitive endurance. Between-group heterogeneity was also non-significant. The GRS funnel plot showed no asymmetry, which Egger’s regression confirmed (intercept = –2.41; 95% CI two-tailed –8.21 to 3.39; two-tailed *p* = 0.36).

### Left Ventricular Twisting Mechanics

#### Twist

**Fig. 3:**
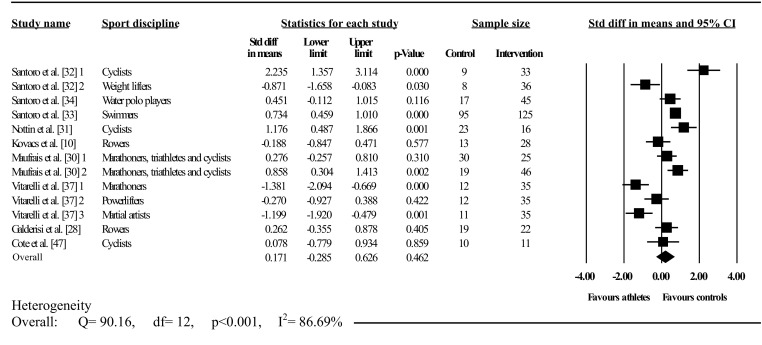
Forest plot showing meta-analysis of overall athlete–control differences in left ventricular twist. *Closed square* study effect size; the size of the symbol and CIs represent study weight and precision, respectively, in the meta-analysis, *closed diamond* overall summary effect, *diamond width* represents overall summary effect precision, *CI* confidence interval, *1*, *2*, and *3* denote multiple athlete–control comparisons from the same study

**Fig. 4:**
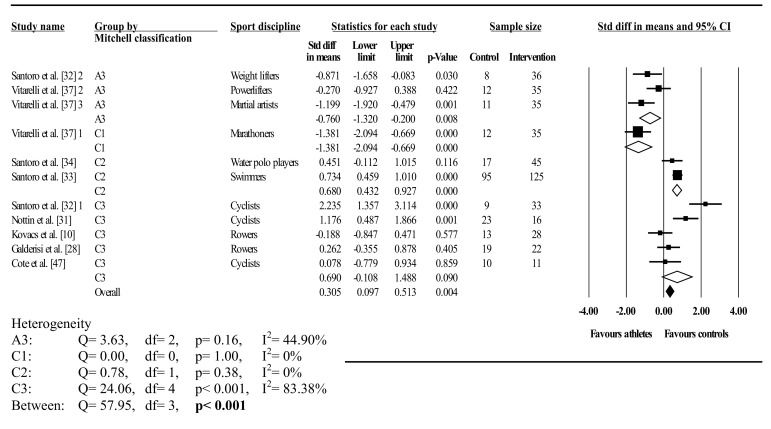
Forest plot showing meta-analysis of athlete–control differences in left ventricular twist categorised by Mitchell’s classification. *Closed square* study effect size; the size of the symbol and CIs represent study weight and precision, respectively, in the meta-analysis, *closed diamond* overall summary effect, *open diamond* overall summary effect within category, *diamond width* represents overall summary effect precision, *A3* high static, low dynamic, *C1* high dynamic, low static, *C2* high dynamic, moderate static, *C3* high dynamic, high static, *CI* confidence interval, *1*, *2*, and *3* denote multiple athlete–comparisons from the same study

**Fig. 5:**
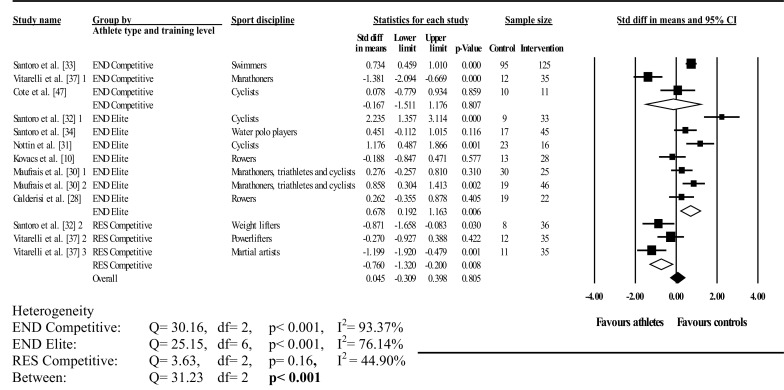
Forest plot showing meta-analysis of athlete–control differences in left ventricular twist using traditional categorization and athlete training level. *Closed square* study effect size; the size of the symbol and CIs represent study weight and precision, respectively, in the meta-analysis, *closed diamond* overall summary effect, *open diamond* overall summary effect within category, *diamond width* represents overall summary effect precision, *CI* confidence interval, *END* endurance, *RES* resistance, *1*, *2*, and *3* denote multiple athlete–control comparisons from the same study

Figures 3, 4 and 5 illustrate athlete–control comparisons and heterogeneity statistics overall, based on Mitchell’s classification and the traditional categorization with training level.

Twist was analysed overall and in A3 (low dynamic, high static), C1 (high dynamic, low static), C2 (high dynamic, moderate static), C3 (high dynamic, high static), elite endurance, competitive endurance, and competitive resistance athlete groups compared with controls. Overall, LV twist did not differ between athletes and controls; this was accompanied by significant and highly inconsistent between-study heterogeneity. Mitchell’s classification showed significantly greater twist in athletes than in controls in A3 and C1. In contrast, twist was significantly less in athletes than in controls in C2, with no differences found in C3. Between-study heterogeneity was non-significant with low inconsistency in A3, C1, and C2. Conversely, significant heterogeneity and high inconsistency occurred in C3; similarly, between-group heterogeneity was also significant.

Traditional categorization showed elite endurance athletes had less twist than controls, whereas competitive resistance athletes had more twist than controls, with no athlete–control differences in competitive endurance athletes. Heterogeneity was significant in both dynamic groups with high inconsistency, whereas resistance competitors showed non-significant heterogeneity with low inconsistency. Further, between-group heterogeneity was significant. Seven studies exceeded the funnel plot, although Egger’s test showed symmetry (intercept = –2.89; 95% CI two-tailed –7.57 to 1.77; two-tailed *p* = 0.20).

#### Basal Rotation

Basal rotation was analysed overall and in A3 (low dynamic, high static), C3 (high dynamic, high static), C2 (high dynamic, moderate static), C1 (high dynamic, low static), elite endurance, competitive endurance, and competitive resistance athlete groups compared with controls. No athlete–control differences existed across any comparisons for basal rotation. Heterogeneity was significant, with moderate inconsistency overall. Between-study heterogeneity was non-significant in A3 and C3 with moderate inconsistency but significant in C2 and C1 with moderate and high inconsistency, respectively. Heterogeneity did not differ between groups overall. Traditional categorization showed significant study-to-study heterogeneity in the elite endurance group with high inconsistency but non-significant in the competitive endurance and competitive resistance groups accompanied by low and moderate inconsistencies, respectively. No differences between groups occurred. Three studies were outside the funnel plot; however, Egger’s test showed symmetry (intercept = 0.60; 95% CI two-tailed –2.41 to 3.62; two-tailed *p* = 0.67).

#### Apical Rotation

Apical rotation was analysed overall and in A3 (low dynamic, high static), C3 (high dynamic, high static), C2 (high dynamic, moderate static), C1 (high dynamic, low static), elite endurance, competitive endurance, and competitive resistance athlete groups compared with controls. Overall, athletes did not differ from controls. Study-to-study heterogeneity was significant and inconsistency high. Sporting categorization showed that apical rotation did not differ between athletes and controls in A3 and C1. In contrast, C2 and C3 athletes had significantly less apical rotation than controls. Within-group heterogeneity was non-significant, with low inconsistency in A3, C2, and C3, whereas significant heterogeneity with high inconsistency was found in C1. Significant between-group heterogeneity was found. Traditional categorization with training level showed no differences in competitive endurance and competitive resistance athletes, whereas elite endurance athletes had significantly less apical rotation than controls. Heterogeneity was significant with high and moderate inconsistency in competitive endurance and elite endurance groups, respectively, with low and non-significant heterogeneity in competitive resistance. Significant between-group heterogeneity was found. Four studies lay outside the funnel plot, two either side, and Egger’s regression test proved symmetry (intercept = –1.32; 95% CI two-tailed –4.99 to 2.34; two-tailed *p* = 0.44).

#### Peak Untwisting Velocity

**Fig. 6:**
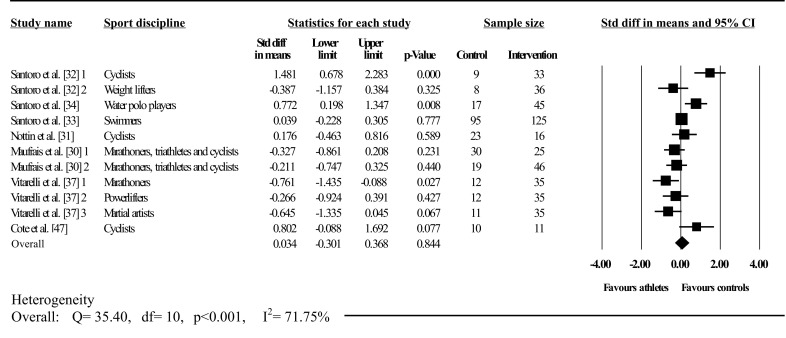
Forest plot showing meta-analysis of overall athlete–control differences in left ventricular peak untwisting velocity. *Closed square* study effect size; the size of symbol and CIs represent study weight and precision, respectively, in the meta-analysis, *closed diamond* overall summary effect, *diamond width* represents overall summary effect precision, *CI* confidence interval, *1*, *2*, and *3* denote multiple athlete–control comparisons from the same study

**Fig. 7:**
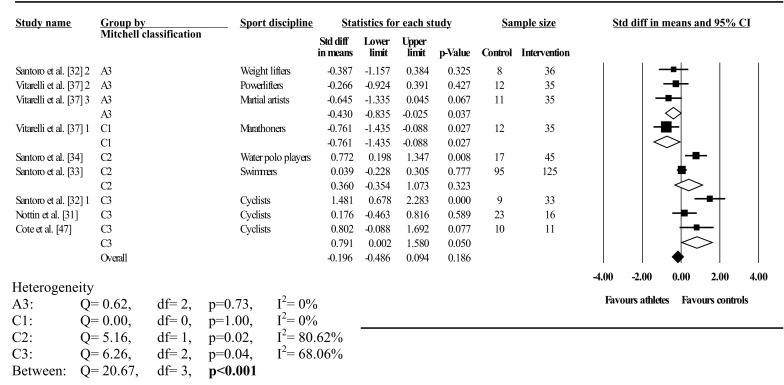
Forest plot showing meta-analysis of athlete–control differences in left ventricular peak untwisting velocity categorized by Mitchell’s classification. *Closed square* study effect size; the size of symbol and CIs represent study weight and precision, respectively, in the meta-analysis, *closed diamond* overall summary effect, *open diamond* overall summary effect within category, *diamond width* represents overall summary effect precision, *A3* high static, low dynamic, *C1* high dynamic, low static, *C2* high dynamic, moderate static, *C3* high dynamic, high static, *CI* confidence interval, *1*, *2*, and *3* denote multiple athlete–control comparisons from the same study

**Fig. 8:**
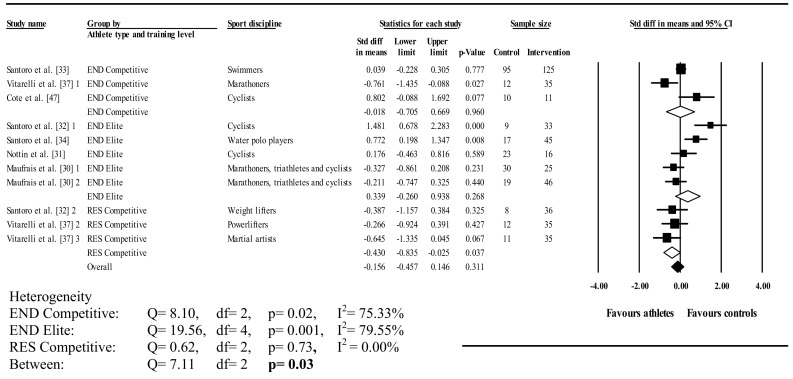
Forest plot showing meta-analysis of athlete–control differences in left ventricular peak untwisting velocity using traditional categorization and athlete training level. *Closed square* study effect size; size of symbol and confidence intervals represent study weight and precision, respectively, in the meta-analysis, *closed diamond* overall summary effect, *open diamond* overall summary effect within category, *diamond width* represents overall summary effect precision, *CI* confidence interval, *END* endurance, *RES* resistance, *1*, *2*, and *3* denote multiple athlete–control comparisons from the same study

Figures 6, 7 and 8 illustrate athlete–control comparisons and heterogeneity statistics overall, based on Mitchell’s and traditional classifications for PUV. PUV was analysed overall and in A3 (low dynamic, high static), C3 (high dynamic, high static), C2 (high dynamic, moderate static), C1 (high dynamic, low static), elite endurance, competitive endurance, and competitive resistance athlete groups compared with controls. Pooled analysis demonstrated PUV did not differ between athletes and controls overall; heterogeneity between studies was significant and moderately inconsistent.

Athletes in A3 and C1 had significantly greater PUV and athletes in C3 had significantly less PUV than controls. There were no differences in C2. A3 and C1 showed non-significant heterogeneity with low inconsistency; C2 and C3 displayed significant heterogeneity with high and moderate inconsistencies, respectively. Further, there was significant between-group heterogeneity. There was no effect when using traditional categorization on PUV in both endurance (elite and competitive) groups, however, both  showed significant heterogeneity with high inconsistencies. In contrast, resistance competitive athletes had significantly greater PUV than controls. Heterogeneity in competitive resistance groups was non-significant with low inconsistency. Heterogeneity was significant between groups.

Athletes had significantly greater UTR than controls (*d* = –0.64; 95% CI –0.99 to –0.30; *p* < 0.001); whereas no differences were observed for PUV (*d* = 0.03; 95% CI –0.30 to 0.37; *p* > 0.05). Within-group heterogeneity in the UTR group was non-significant with low inconsistency (*Q* = 5.10; *I* ^2^ statistic = 21.59%; *p* > 0.05). In contrast, significant heterogeneity with moderate inconsistency was found in the PUV group (*Q* = 35.40; *I* ^2^ statistic = 71.75%; *p* < 0.001). Similarly, UTR versus PUV heterogeneity was significant (*Q* = 13.82; *p* < 0.001).

Three studies lay outside the PUV funnel plot; however, symmetry was proved by Egger’s regression test (intercept = 0.41; 95% CI two-tailed –3.25 to 4.06; two-tailed *p* = 0.81).

### Meta-Regressions

Table 4 provides all meta-regression associations with strain and LV mechanical parameters. LVMi was indexed to body surface area [23, 32–34, 53] and height [28, 30]; two studies did not detail what LV mass was indexed to [36, 54]. LVMi showed significant positive relationships with GLS. Significant negative associations were also observed between SBP and PUV and GRS with age in the overall sample. No further significant associations were found.

**Table 4:** Meta-regressions of athlete-control differences in left ventricular strain and twisting mechanics with covariates

Covariate parameter	Number of studies	Cochran’s *Q*	SE	*β*	95% CI	*p* value
Global longitudinal strain						
Age	14	1.03	0.04	–0.04	–0.11 to 0.03	0.31
HR	13	0.33	0.03	0.01	–0.04 to 0.07	0.57
SBP	14	1.57	0.03	–0.03	–0.09 to 0.02	0.21
DBP	14	0.20	0.03	0.01	–0.04 to 0.07	0.66
LVMi	10	5.41	0.005	0.01	0.002 to 0.02	0.02
Basal circumferential strain						
Age	7	0.02	0.03	–0.003	–0.05 to 0.05	0.90
HR	7	0.86	0.02	–0.02	–0.06 to 0.02	0.36
SBP	7	1.07	0.02	–0.02	–0.06 to 0.01	0.30
DBP	7	2.99	0.03	0.06	–0.01 to 0.12	0.08
LVMi	6	2.52	0.004	0.01	–0.001 to 0.01	0.11
Apical circumferential strain						
Age	7	0.02	0.03	–0.005	–0.07 to 0.06	0.88
HR	7	0.12	0.03	–0.01	–0.06 to 0.04	0.73
SBP	7	0.52	0.03	–0.02	–0.07 to 0.03	0.47
DBP	7	0.22	0.06	0.03	–0.09 to 0.14	0.64
LVMi	6	3.47	0.004	0.01	–0.004 to 0.02	0.06
Global circumferential strain						
Age	7	1.57	0.09	0.11	–0.06 to 0.28	0.21
HR	6	0.48	0.03	0.02	–0.04 to 0.08	0.49
SBP	7	0.20	0.04	0.02	–0.07 to 0.11	0.65
DBP	7	2.60	0.03	0.04	–0.01 to 0.10	0.11
LVMi	4	0.02	0.01	0.001	–0.02 to 0.02	0.90
Global radial strain						
Age	9	4.19	0.04	–0.09	–0.17 to 0.004	0.04
HR	8	0.10	0.02	0.01	–0.04 to 0.05	0.75
SBP	9	0.54	0.02	0.02	–0.03 to 0.06	0.46
DBP	9	0.39	0.02	–0.01	–0.06 to 0.03	0.53
LVMi	5	0.04	0.01	0.002	–0.01 to 0.02	0.84
Twist						
Age	13	0.01	0.05	–0.01	–0.10 to 0.09	0.91
HR	13	1.25	0.04	–0.05	–0.13 to 0.04	0.26
SBP	13	0.08	0.04	–0.01	–0.08 to 0.06	0.77
DBP	13	0.74	0.08	–0.07	–0.23 to 0.09	0.39
LVMi	8	0.35	0.01	0.005	–0.01 to 0.02	0.56
Basal rotation						
Age	14	0.38	0.03	–0.02	–0.08 to 0.04	0.54
HR	14	0.96	0.03	0.03	–0.03 to 0.08	0.33
SBP	14	0.10	0.02	–0.01	–0.05 to 0.04	0.75
DBP	14	0.02	0.05	0.01	–0.10 to 0.12	0.88
LVMi	8	0.07	0.005	–0.001	–0.01 to 0.01	0.79
Apical rotation						
Age	13	0.21	0.04	–0.02	–0.09 to 0.05	0.65
HR	13	0.003	0.03	0.002	–0.06 to 0.07	0.96
SBP	13	0.004	0.04	0.002	–0.07 to 0.07	0.95
DBP	13	0.92	0.06	–0.06	–0.18 to 0.06	0.34
LVMi	8	0.31	0.004	0.002	–0.01 to 0.01	0.58
Peak untwisting velocity						
Age	11	0.18	0.03	0.01	–0.05 to 0.08	0.67
HR	11	1.28	0.03	–0.03	–0.09 to 0.03	0.26
SBP	11	3.95	0.03	–0.06	–0.13 to –0.001	0.05
DBP	11	1.35	0.06	–0.07	–0.18 to 0.05	0.25
LVMi	7	0.84	0.01	0.01	–0.02 to 0.04	0.36

*CI* confidence interval, *DBP* diastolic blood pressure, *HR* heart rate, *LVMi* left ventricular mass index, *SBP* systolic blood pressure, *SE* standard error

## Discussion

The main findings from our study are that, when sporting categorizations are ignored, there are no differences in LV strain and twisting mechanics, besides UTR, in athletes compared with non-exercising controls. However, when athletes are categorized according to the static and dynamic demands of their individual sports using Mitchell’s classifications, differences do emerge, predominantly in twist mechanics. Cardiac twist was greater in athletes in A3 (low-dynamic high-static; weightlifting, martial arts, etc.) and C1 (high-dynamic low-static; distance running, soccer, etc.) than in their untrained counterparts. In contrast, twist was lower in athletes in C2 (high-dynamic moderate-static; swimming, water polo, etc.), which was driven by alterations in apical rotation but not basal rotation. PUV was found to be greater in athletes in A3 (weightlifting, martial arts, etc.) and C1 (distance running, soccer, etc.) but less than controls for athletes in C3 (high-dynamic high-static; rowing, cycling, etc.). Additionally, using the traditional categorization, endurance athletes showed a trend towards reduced LV twist compared with controls; therefore, training level subdivisions revealed that elite endurance athletes demonstrated significantly less twist than controls, which was accompanied by lower apical rotation that was not found in competitive endurance athletes. In contrast, competitive resistance athletes showed increased twist and subsequent PUV compared with controls. Athletes demonstrated significantly increased UTR compared with controls. Finally, LVMi, a measure of cardiac adaptation, was significantly and positively associated with GLS.

This is the first meta-analysis to investigate the influence of exercise training on 2D STE-derived LV mechanics. These data provide further understanding of athlete–control differences in LV STE-derived indices.

### Global Longitudinal Strain

Collectively, GLS did not differ in athletes compared with matched controls. The lack of overall effect may be explained by significant inter-study heterogeneity. Further, subgroup analyses indicated GLS in athletes remained unchanged, which suggests GLS does not alter in trained athletes, at least at rest. Other work has demonstrated that GLS remained unchanged during incremental exercise after the initial workload (20% maximum aerobic power) [55]. Further, longitudinal strain did not change during afterload elevated exercise using isometric hand-grip [56]. GLS has shown limited augmentation during exercise, whereas other myocardial STE parameters (i.e., circumferential strain, LV twist mechanics) may play a more pivotal role in augmenting myocardial function during effort. Thus, changes in GLS may not be necessary in athletic populations.

Despite the lack of differences between controls and athletes in this meta-analysis, studies have demonstrated increased longitudinal strain following exercise training programs ranging from 3 to 39 months in duration [57–61]. If longitudinal strain is altered in athletes, it is likely to be increased, since a reduction is not a common feature of the athlete’s heart [39]. Lower GLS may be attributed to predominately unhealthy patients; healthy subjects regardless of training status (i.e., both trained and untrained) possess normal longitudinal strain at rest, observed to be –19.7% (95% CI –18.9 to –20.4) in a previous meta-analysis [62]. Indeed, a review suggested that individual athletes with significantly reduced GLS accompanied by LV hypertrophy should be carefully evaluated [39]. This meta-analysis supports those suggestions given that exercise training appears to have little or no effect on GLS, so it is not decreased in athletes.

Since GLS is measured on a negative scale, the positive association between LVMi and GLS indicates that GLS decreased as LVMi increased in athletes relative to controls. The interaction is indicative of reduced GLS with increasing cardiac hypertrophy, suggesting enhancement of a reserve with increasing relative cardiac mass. However, any such functional reserve may be small given the lack of overall difference in GLS between athletes and controls.

In terms of cardiovascular disease, reduced GLS has been demonstrated in hypertensive and hypertrophic cardiomyopathy populations [25, 28, 34, 63, 64], supporting the contention that reductions in GLS may be maladaptive and associated with cardiovascular disease abnormalities. Therefore, reduced longitudinal strain could be considered an early sign of dysfunction, such as myocardial fibrosis, which is associated with a 3.4-fold increased risk of major adverse events [65]. In patients with hypertrophic cardiomyopathy with normal conventional systolic and diastolic function, GLS was significantly lower in those with late gadolinium enhancement (a quantifiable tool to assess myocardial fibrosis) than in those without [66]. This suggests a link between the extent of fibrosis and GLS, and thus GLS may be considered a sensitive superior marker for early detection of dysfunction in the absence of global abnormalities. This also supports the current guidelines that recommend GLS as a reproducible and feasible tool for clinical use; it can provide incremental data over traditional measures of systolic function [67].

Consideration of these findings and our growing understanding of the changes in longitudinal strain under various conditions may prompt the translation of GLS into clinical practice to aid in the detection of adverse remodeling and distinguishing pathological from physiological functional remodelling prior to major cardiovascular events.

### Circumferential Strain

Neither basal nor GCS demonstrated significant athlete–control differences; however, there was a trend for reduced ACS in athletes relative to controls. Circumferential strain progressively increases with exercise [55], while other work has shown that ACS increases during exercise but BCS remains unchanged [68]. Since the apex permits a more dynamic behavior than the base when the myocardium is subjected to physiological demands, and thus may have a greater reserve to respond to exercise [49], it is possible that any adaptive reductions in ACS at rest may contribute a functional reserve that could become available for utilization during effort to enhance GCS.

ACS and BCS are not influenced by sport-specificity; no alterations were observed following Mitchell’s categorization. Conversely, GCS was significantly reduced in B3 (body building, wrestling, etc.). This finding comes from a sole study using trained body builders [23], so this finding should be interpreted with caution. Although we excluded articles involving the use of performance-enhancing drugs, other work has demonstrated significantly diminished ACS in anabolic steroid users [69]. Any undisclosed use of anabolic steroids may have contributed to the observed GCS reductions.

Competitive endurance athletes demonstrated greater BCS than controls. Despite this observation, the summary effect was heavily influenced by a single investigation (relative weight –91.07%), the only study to show a significant effect and containing a large sample size and high precision [33]. Consequently, whether competitive endurance athletes have greater BCS remains unknown, and further studies with large populations are warranted to provide further insight into the initial observations.

### Global Radial Strain

GRS did not differ between athletes and controls during any comparisons, whether overall or with Mitchell’s or traditional classifications. Further, no individual studies showed significant effects between athletes and controls, and the study sample was considered homogenous. GRS is a surrogate measure of cardiac contractility as it represents strain in a plane orthogonal to the direction of sarcomere shortening. In addition, previous analysis of GRS has shown it to be the most variable strain measure, with a test–retest reproducibility of 19% coefficient of variation [70] and measurement variability of 35.9% [71]. The large variance inherent in the measurement of GRS may explain the lack of athlete–control differences observed to date given the level of variability within the measure itself, potentially due to out-of-plane motion [70].

### Left Ventricular Twisting Mechanics

#### Twisting

Overall twist did not differ between athletes and controls, accompanied by a large and highly significant heterogeneous sample. Following Mitchell’s classification, the present data showed multiple intriguing observations. A3 (weightlifting, martial arts, etc.) and C1 (distance running, soccer, etc.) athletes had greater twist than controls, whereas twist in C2 athletes was less than in controls. Although C1 (distance running, soccer, etc.) demonstrated significantly greater twist in athletes, these findings came from a single study, whereas A3 (weightlifting, martial arts, etc.) was determined to be homogenous from multiple studies.

Despite literature frequently disputing concentric morphological adaptations in resistance-trained athletes [20, 21], the findings of this meta-analysis show that functional STE-derived alterations exist. Afterload conditions may partly explain greater twist in the high-static low-dynamic sporting disciplines. Unlike C2 athletes, compensatory twist in A3 athletes could become necessary to overcome aortic pressure, providing a more forceful contraction for ejection. LV twist progressively increased with advancing levels of afterload in patients with hypertension or aortic stenosis [34]. In this meta-analysis, we observed a trend towards significantly greater apical rotation in A3 (weightlifting, martial arts, etc.) athletes. Experimental studies inducing afterload with isometric hand-grip exercises have shown impaired LV twist via reductions in apical rotation [48, 56]. Repeated exposure to acute afterload increases may lead to chronic adaptations in twist to maintain systolic function mediated by increased baseline apical rotation, a compensatory mechanism in high-static low-dynamic (A3) athletes. Coupled with enhanced afterload, the unchanged [21, 32, 72, 73] or modestly increased [22, 74] LV chamber size typically associated with concentric morphological adaptations, with unchanged end-diastolic volume [74], could further accentuate twist to eject a stroke volume adequate for supporting baseline cardiovascular functioning. Additionally, geometry alterations with greater wall thickness, relative to short-axis cavity dimensions, may provide an explanation for greater twist. It is well established that the longer lever arm of the subepicardium compared with the subendocardium dominates the direction of rotation because of its larger radius [31]. Other work has associated increased wall thickness with greater apical rotation and thus LV twist [75]; amplifying the distance between the two contour layers as a result of thicker walls could cause even greater dominance of epicardial rotation and potentially explain the increased twist in highly static low-dynamic athletes.

Lower twist and apical rotation in C2, which conflicts with that observed in A3 athletes, could be explained by LV volume changes and chronic adaptations. Both studies recruited athletes from water-based sports (water polo players [34] and swimmers [33]) and exhibited increased LV internal diameter [33, 34] and end-diastolic volume [33]. Underwater exercise induces greater hydrostatic pressure, central volume, and thus preload [76], which may contribute to the observed enlargements [77]. Although increases in LV twist with preload manipulation have been observed following saline administration [78, 79], which artificially increases LV end-diastolic volume and internal diameter and activates the Frank–Starling mechanism, this may not cause the same twisting responses as pre-existing LV structural alterations brought about by training-induced physiological adaptations. Greater LV chamber adaptations to training may facilitate a functional reserve in systolic mechanics. In support, two longitudinal studies of relatively short duration (acute) endurance exercise training (3 months [61] and 6 months [57]) led to increased LV twist and apical rotation. More recently, a chronic maintenance program (36 months) showed LV twist and apical rotation regressed to baseline levels [60]. Given the aforementioned influence that heightened preload has on twist [78, 79], facilitated by the Frank–Starling mechanism, these responses following the acute phases may be mediated by greater plasma and thus volume expansion, leading to larger end-diastolic volumes [60]. In contrast, the morphological adaptions observed consequent to the chronic phase, including increased LV length and wall thickness, may therefore accommodate heightened blood volume and contribute to reduced twist. LV sphericity index and twist are related in a parabolic manner [75]; with increased LV length, demonstrating a more elliptical ventricle, chronically trained athletes may represent the lower right side of the curve whereby twist will become reduced, possibly because of alterations in myocardial fiber angle, as shape and fiber orientation are closely associated [80]. Irrespective of mechanistic underpinning, these longitudinal observations suggest that cardiac twisting profiles follow a phasic response to training in athletes, which therefore may also assist in explaining potential causes of heterogeneity as found in this meta-analysis.

When categorized according to traditional methods, alongside the level of athletic accomplishment, the elite endurance group demonstrated significantly reduced twist, and no differences were seen in the competitive endurance group compared with controls. Further, apical rotation was reduced in elite athletes, but basal rotation did not differ. The apex is suggested to be more ‘free’ than the base because it is more elastic and not tethered to the right ventricle, which may therefore permit more rotation at the apex [81]. In laboratory-based settings, literature has frequently documented greater apical augmentation with submaximal exercise than at the base [49, 55, 82]. This is potentially because of its greater β-receptor density and responsiveness to adrenergic stimulation [83], greater augmentation in response to heightened preload [79], or a combination of both. The apex is suggested to have a greater functional reserve to respond to exercise than the base [49] and, considering the superior sensitivity of the apex with the onset of increased cardiovascular demand, it is unsurprising that the more caudal region of the myocardium presents a baseline adaption. Along with the potential cardiac geometry changes and their influential effects on twist mechanics, LV twist is lower with a decreased resting HR [31] and following exercise training; changes in sympathovagal balance cause decreases and increases in sympathetic and parasympathetic activity, respectively [32]. Greater β adrenergic receptor concentration within the apex might explain reduced apical rotation and twist due to heightened sensitivity to Ca^2+^ release and uptake [33], whereby normal functioning is maintained with decreased systolic twist at rest. Another mechanism concerns alterations in the myocardial fibers; elite athletes may present greater contractility of the subendocardial layer, thereby reducing the net twist. In contrast, reductions in both the inner and the outer layers may also partly explain reduced LV twist and thus apical rotation, as demonstrated by Nottin et al. [31] in elite cyclists.

Dynamic exercise induces elevations in preload, and consequently exercise performance may benefit from greater twist during effort, especially in elite athletes. It is commonly known that endurance athletes demonstrate functional reserves in basic physiological measures including HR, blood pressure, etc., at rest compared with untrained populations. Given this meta-analysis found reduced twist in elite endurance athletes, it may be plausible that there exists a necessary functional reserve of apical rotation and thus twist to attain a superior level of sporting performance. Nevertheless, more research is still required to establish the ‘true’ nature of reduced twist mechanics in elite athletes and its interaction with global LV function; this is likely to require study of twist mechanics during exercise. For example, LV twist plateaued during incremental exercise at moderate intensities, which is a suggested mechanical limitation to stroke volume in recreationally active individuals [49]. LV twist is linearly related to stroke volume [49, 78] and, since stroke volume progressively increases to maximum in endurance athletes [84], it is plausible that reduced resting twist in elite endurance athletes may facilitate continual LV output to high-intensity exercise. However, in light of the available literature, this remains in contention. Clarification will require determining whether the baseline physiological adaptation is because athletes possess a functional reserve that may be called upon during exercise. Indeed, limited work indicates that, even in non-athletic individuals, apical rotation was lower at rest and during submaximal exercise (40% peak power output) in those with high aerobic fitness than in those with moderate aerobic fitness [68]. This reduction may be indicative of a functional reserve even during submaximal exercise and additionally supports that twist may have capabilities of increasing beyond moderate intensities. Further studies in elite endurance individuals will aid in bridging the gap between global traditional measures of systolic function and ‘novel’ measures (twist mechanics).

Despite numerous studies with competitive endurance athletes reporting increased structural adaptions [33, 37, 53], the lack of overall effect in twist mechanics could suggest that structural adaptions precede those of functional STE-derived indices in competitively trained athletes. However, in two of the studies, LV twist differed significantly between athletes and controls but in opposing directions [33, 37]; therefore, further data are necessary to expose the large heterogeneity in studies with competitive athletes to further establish the dose–response relationship between exercise training and twist mechanics. However, from the literature to date, and thus the findings of this meta-analysis, alterations in LV twist appear to be attributed to elite-level populations performing predominantly highly dynamic exercise. Conversely, competitive resistance athletes showed a compensatory increase in twist compared with controls. No elite resistance studies were available in this meta-analysis, which prevented a direct comparison between training levels in resistance-trained athletes. Therefore, whether athletes of a greater training level within static disciplines demonstrate a further increased twist than seen in the competitive performers remains unknown.

#### Untwisting

Untwisting velocity was not different in athletes compared with controls overall. Similar to LV twist, heterogeneity was significant, but sport-specific alterations were found. The A3 (weightlifting, martial arts, etc.) and C1 (distance running, soccer, etc.) athletes showed greater PUV than controls, suggesting a systolic–diastolic coupling (i.e., concomitantly increased twist and PUV vs. controls). In contrast, C3 (all cyclists) exhibited significantly reduced PUV. Although the findings of the present meta-analysis did not show a twist–PUV coupling in C3 (rowing, cycling, etc.), a significant reduction in athlete’s twist was apparent when additional LV twist analysis was conducted using the same studies as used for PUV analysis [31, 32, 53] (*p* = 0.05) (data not presented), indicating a systolic–diastolic mechanical coupling (i.e., concomitantly decreased twist and PUV vs. controls).

Stored energy following systolic twist prompts the release of energy within the spring-like titin protein [85] to cause untwisting. Untwisting produces a ‘suction’ effect by creating an intraventricular pressure gradient (IVPG) [82]; the ability to create this gradient facilitates passive filling, providing superior diastolic function [86]. Lower ventricular pressure facilitates passive LV filling with low atrial pressures [87], and the relationship between IVPG and untwisting has been shown to be positive [88]. The LV twist/untwist interaction is also documented as positive [88]; thus, the increased twist found in A3 (weightlifting, martial arts, etc.) may explain greater PUV as a compensatory mechanism to enhance filling.

Reduced PUV may be due to reductions in twist at rest, with the myocardium requiring less twist and thus untwisting to attain sufficient resting cardiovascular function [38], a suggested reserve mechanism for exercise [32, 38]. Lower HR, elongated diastolic filling periods consequent to preserved LV pressure decay (tau), and diastasis may facilitate reduced PUV. A strong negative association has been observed between untwist and tau in dogs (*r* = –0.66, *p* < 0.0001) [88]. Greater parasympathetic activity could preserve untwisting until inotropic stimulation occurs during exercising conditions. In support, progressive administration of dobutamine caused proportional increases in twist and PUV, whilst tau progressively decreased and HR remained unchanged from baseline [88]. As with systolic twist, further research on the untwisting responses in athletes, both at rest and during exercise, will help establish whether a functional reserve in PUV is present in high-dynamic high-static sports, as suggested by the results of this meta-analysis.

Limited data are available on diastolic twist mechanics following longitudinal exercise training. Weiner et al. [61] reported on university athletes following 3 months of rowing training; they exhibited early diastolic PUV and increased %untwist during isovolumic relaxation time (IVRT), with no further changes in early diastolic PUV after the ensuing chronic maintenance program—unlike twist, which regressed to baseline [60]. The initial increase probably occurred due to volume expansion, since other work has demonstrated the preload dependency of early diastolic PUV [60, 79]. However, after the chronic phase, adaptive hypertrophic remodeling occurred. Therefore, the preserved supernormal diastolic function may reflect an intrinsic functional adaption in untwisting mechanics. Additional mechanistic contributions for altered mechanics other than HR and sympathovagal balance are suggested. Changes in the titin isoforms could be responsible for potential compensatory increases and functional reserves in rotational mechanics, as found in this meta-analysis. Titin, a bidirectional myocardium filament plays a crucial role in storing forces necessary for early diastolic function [89]. Different spring compositions alter passive stiffness; this variation influences passive and restoring forces. Methawasin et al. [90] showed that greater titin compliance attenuated the Frank–Starling mechanism, whereas stiffer isoforms showed greater length-dependent activation. Diastolic function is influenced by increases in titin-based compliance, which manifests in increased LV chamber compliance [90]. Shifts to more elastic isoforms could increase the quantity of energy released during early diastole prior to MVO [30], as was found in elite endurance athletes, who demonstrated significantly greater peak kinetic energy during early diastole [91]. Titin phosphorylation and isoform shifts have shown alterations with cardiac disease [92]; adjustments in athletes may partly explain divergent athlete–control differences in LV twist mechanics. Findings from this meta-analysis showed greater UTR in athletes, suggesting facilitation for early LV filling, and other investigations have shown greater UTR [37], %untwist during IVRT [30], and shorter time to PUV [29] in athletes with no differences in PUV compared with controls. Thus, athletes may present noticeable enhancements in diastolic function as measured during the earliest phases of relaxation (i.e., before MVO) even when PUV differences are absent. During resting conditions in those with normal diastolic function, alterations in PUV may not obviously differ between trained and untrained individuals, potentially due to the long durations of diastole at rest, thus PUV may become a more influential parameter for assessment when the filling period diminishes, i.e., during exercise. Due to the significant proportion of untwist that occurs during the IVRT (~50 to 70%) [13], parameters reflecting the earliest phases of relaxation may be considered more sensitive markers of diastolic function when distinguishing trained and untrained populations. Athletes often have normal or superior global diastolic function as measured using conventional markers such as the E, A, and E/A ratio [22, 93]. These observations may be underpinned by early untwisting, allowing the generation of a sufficient pressure gradient and, thus, measurements of untwisting mechanics before mitral inflow may provide a precursor to the traditional well-established parameters. However, it is clear that further substantiation is required in athletic populations to fully understand how exercise training influences untwisting mechanics, with particular interest in potential differences between UTR and PUV. Consequently, until untwisting mechanics are better understood, conventional global measures of diastolic function may remain more suitable parameters to differentiate pathology and physiology in athletic patients.

Following meta-regression analysis, as SBP increased, the difference in PUV effect size between athletes and control diminished. This association is suggestive of increased afterload exerting influences on LV twist mechanics, thus reducing the functional reserve in diastolic function.

### Study Limitations and Future Studies

Several limitations within this meta-analysis must be addressed. The first concerns the use of the random-effects model, which does not assume all studies are equal but that the true effect varies between studies, and the analysis estimates the mean distribution of effects [94]. Smaller studies become more influential and reduce the relative weight of larger studies, to account for the within-study variability and ‘balance’ the outcome [94]. Between-study variances may be influenced by echocardiographic inconsistencies during image acquisition and analysis. LV twist mechanics have greater variability (apical rotation [8–50%] [31, 49, 55, 70, 95], basal rotation [5–21%] [55], twist [10–20%] [49, 70, 96], and PUV [26%] [96]) than longitudinal and circumferential strain (<8%) [55, 70]. When the high variability of STE-derived measures is compounded by small sample sizes, as is the case in several studies included within this meta-analysis, it is likely that studies are underpowered to detect subtle differences between athletes and controls. This may explain why, in some cases, we observed only minimal differences between athletes and controls. Moreover, when assessing the apex, progressive caudal transducer movement is associated with increased apical rotation [95, 97]. Given that the present meta-analysis indicates alterations in LV twist with concomitant changes in apical rotation, the importance of consistent and accurate apical acquisition, allied to consistent and accurate reporting of the location of apical measures, in reducing study-to-study heterogeneity is clear. Publication bias only occurred for BCS, therefore findings from this meta-analysis for all remaining measures suggest an unbiased thorough collection of sample studies representative of completed literature. Nevertheless, in common with many systematic reviews, it is possible that we have missed some data, particularly from studies published in languages other than English.

Our use of Mitchell’s classification, although widely accepted as a method of categorizing sporting activities, has several inherent limitations. First, sporting categorization is not position specific, which has implications for team games. For example, the dynamic and static loading experienced by a goalkeeper and midfielder in soccer should not be considered equal. Second, the model classifies the activity, not the athlete. This may be an issue, particularly in elite-level sport where athletes likely undertake additional strength and conditioning training to supplement competition training. Clearly, the possibility exists that this may alter the dynamic and static components and thus cardiac loading [24]. In conjunction with our findings, we suggest training level be considered when interpreting study findings. Further, when including two or more athlete groups, studies should obtain participants of a similar competition standard and training level.

Furthermore, the inclusion criteria for this analysis included healthy males aged 18–45 years, so our findings cannot be extended to female, older (>45 years) or younger (<18 years) populations. A broad age range was adopted to maximize study inclusion; however, no associations were evident between age and STE-derived measures following meta-regression analysis in twist mechanics. Therefore, the study age range used in this meta-analysis can be considered homogenous and is unlikely to account for some of the between-study heterogeneity. Although still controversial, LV systolic twist mechanics and rotations appear to be sex independent [42, 44, 53], whereas other work has suggested sex influences GLS [26, 41, 42]. However, twist mechanics are repeatedly documented as affected by age [44–46], therefore to eliminate any confounding factors and for homogeneity purposes we suggest that future studies recruit single-sex age-matched groups.

We analysed data on global indices of LV strain. Therefore, whether athletes develop regional alterations in specific segments of the myocardium, where global differences were undetected, is beyond the scope of this study; future work may wish to explore this potential.

Training-level subdivision was only conducted when using the traditional method of categorization, since additional division of elite and competitive athletes when using Mitchell’s classification would have resulted in only a few studies within each groups, as 18 categories were possible. The effect of training duration and protocols may also be important. In particular, this meta-analysis did not consider the stage or duration of training, and most studies did not report the training phase of athletes during data collection. Given the possibility of a phasic response of exercise training on LV twist [60], this may account for some of the between-study heterogeneity observed in this meta-analysis. Future studies should acknowledge and consider the phase, volume, and intensity of training, as well as the time within a season that athletes are tested. Accordingly, we recommend that more longitudinal studies are conducted, which may eliminate much of the heterogeneity observed between existing observational studies. More studies are required to establish additional sources of between-study heterogeneity. For example, Oxborough et al. [98] recently used novel strain–volume/area loops to study simultaneous strain and structure, suggesting differences in peak longitudinal strain are a reflection of chamber size following the finding of normalized strain for% end-diastolic volume. Future work may wish to explore the interaction between LV mechanics and volume/area in chronically trained athletes. Further to this, few studies have investigated the effect of body size on LV mechanics. Since it is currently recommended that traditional structural measures be scaled to body surface area [67] to enable direct comparisons, more studies are required to understand the influence of body size and thus scaling on LV mechanics.

We did not account for inter-vendor differences, and it is possible that vendor differences in the algorithms and thus analysis of speckle-tracking measurements may account for some heterogeneity observed, as previously acknowledged [13, 74]. Therefore, these differences should be considered when interpreting associated LV mechanics data.

Some recent attention has been directed towards strain of the right ventricle (RV) following prolonged exercise [99]. Although the focus of this meta-analysis is primarily related to LV mechanics, it is important to acknowledge the possible impact of training on the RV. It is well established that athletes develop enlargement of the RV, albeit in the presence of normal systolic and diastolic function as determined by conventional indices such as RV fractional area change and tricuspid plane systolic excursion (TAPSE). In view of this, few studies have attempted to define RV longitudinal regional and global strain [100–104]. Teske et al. [104] demonstrated a reduced basal systolic strain rate in athletes with a dilated RV; others have demonstrated values similar to those in non-athletic controls [102]. These heterogeneous findings are likely a consequence of variable athlete demographics similar to those seen in studies of the LV. The parallel interaction of RV size and function on the LV is equally important and may, in part, explain some of the findings presented in this review, particularly in the septal regions and ventricular insertion points. It is apparent that further work to systematically explore the literature in this area is warranted.

The available literature also has limitations. Within the included studies, limited reporting of anthropometric data prevented additional meta-regression or moderator analyses, which may have further identified sources of heterogeneity between comparisons. Future investigators may wish to consider reporting basic anthropometric data along with cardiac data associated with the athlete’s heart.

A further important limitation is the different criteria used to classify the control or non-trained group. Subjects’ level of exercise ranged from untrained and sedentary [10, 32–34, 37] to exercising <2 h/week [54], exercising <3 h/week [23], and recreationally active (3.9 ± 1.5 days/week) [53]. Given these differences between dynamic training levels, it is important that control groups are as homogenous as possible and preferably sedentary, which may eliminate some between-study heterogeneity and provide more clarity on the effects of exercise training on LV mechanics. However, when recruitment of completely sedentary participants is not possible, studies should report data detailing exercise volume and intensity.

From the available literature within this meta-analysis, only one study attempted to differentiate sports based on the variation of static and dynamic components [37]. However, the intermediate group (martial artists), considered by the authors as combined strength and endurance, is actually classified as a high-static low-dynamic sport according to Mitchell’s classification. Consequently, we recommend that future studies incorporate a spread of athlete types alongside Mitchell’s framework as opposed to dichotomous athlete grouping to expand on the sport-specific alterations in cardiac twist mechanics.

When investigating LV untwist and consequently diastolic function, studies should assess both UTR (early diastole) and PUV as separate parameters to provide more useful insights into athletes’ diastolic responses at various timing events, which will further enable a greater understanding of the relative importance of each measure, especially during resting conditions. To date, only one study [37] has done this. Additionally, the measurement point of diastolic markers should be more clearly identified, which may eradicate some heterogeneity via the use of consistent terminology.

Given the large heterogeneity observed throughout, future research is warranted while considering sporting discipline, training level, and covariates as identified from this meta-analysis. At present, without additional knowledge regarding the direction of alterations in LV strain and twist mechanics, aside from GLS, the findings of this analysis support the suggestion that it may not be feasible to use baseline LV mechanics clinically to differentiate pathological and physiological remodeling [37].

## Conclusion

Apart from UTR, when sporting categorization was not implemented, no differences between trained athletes and untrained healthy controls existed in any LV STE-derived parameters. However, GLS may have the potential to become a promising parameter to aid in the diagnosis between pathological and physiological remodeling because exercise training has little to no effect. This meta-analysis has shown that 2D STE may be used to distinguish cardiac functional changes when taking athletic type and training level into consideration. Elite-level endurance athletes demonstrated reduced LV twist accompanied by lower apical rotation at rest, which may not be present in competitive-level athletes. Thus, it is plausible that a dose–response relationship may exist between endurance exercise training level and alterations in LV twist. Athletes exposed to differing cardiac loading associated with the dynamic and static components of sports possess divergent twisting mechanical profiles, with low-dynamic high-static sports presenting a potential compensated increase in twist. Further, PUV was greater in low-dynamic high-static sports but lower in high-dynamic high-static sports. The results of the meta-regressions suggest that relative cardiac size and hemodynamic loading conditions should be considered when interpreting data from future studies. Each of these covariates may also partly explain some inter-study heterogeneity and inconsistency.

LV twist mechanics depend on sporting type or training level or a combination of both. Suitable athlete categorization using both traditional and contemporary methods have proved to be potentially useful tools for extrapolating LV twisting mechanics in athletes. Therefore, future studies should consider sporting type and athlete training level simultaneously. With the promising use of 2D STE coupled with improved data reporting leading to homogenous athlete and control samples, greater certainty regarding alterations in STE-derived LV mechanics consequent to exercise training can be elucidated.
